# Efficient Adsorption of Methylene Blue by Polyaminocarboxylated Modified Hydrochar Derived from Sugarcane Bagasse

**DOI:** 10.3390/molecules30071536

**Published:** 2025-03-30

**Authors:** Xi Liu, Feng Zhou, Changrong Shi, Jerome Ramirez, Zhihua Liu, Fangxue Hang, Caifeng Xie

**Affiliations:** 1 College of Light Industry and Food Engineering, Guangxi University, Nanning 530004, China; xiliu_520@163.com (X.L.); 18573202226@163.com (F.Z.); 2216301031@st.gxu.edu.cn (Z.L.); hangfx@163.com (F.H.); 2 Faculty of Chemical Engineering, Kunming University of Science and Technology, Kunming 650500, China; c.shi@kust.edu.cn; 3 School of Mechanical, Medical and Process Engineering, Science and Engineering Faculty, Queensland University of Technology, Brisbane, QLD 4000, Australia; 4 Engineering Research Center for Sugar Industry and Comprehensive Utilization, Ministry of Education, Nanning 530004, China; 5 Provincial and Ministerial Collaborative Innovation Center for Sugar Industry, Nanning 530004, China; 6 Academy of Sugarcane and Sugar Industry, Guangxi University, Nanning 530004, China

**Keywords:** sugarcane bagasse, polyaminocarboxylated modified hydrochar, adsorption, methylene blue, DFT simulation

## Abstract

Sugarcane bagasse (SCB) was transformed into polyaminocarboxylated modified hydrochar (ACHC) by hydrothermal carbonization (HTC), which was then followed by activation, etherification, amination, and carboxylation successively. ACHC was systematically characterized, and batch adsorption studies were used to assess its methylene blue (MB) adsorption capacity. Adsorption was analyzed by adsorption isotherm models, the adsorption mass transfer model, and the adsorption thermodynamics model. Density functional theory (DFT) was utilized to explain adsorption mechanisms. The findings demonstrated the adsorption was one type of endothermic, spontaneous, and homogenous monolayer adsorption with intra-particle diffusion, containing both chemical and physical adsorption, involving electrostatic attraction, hydrogen bonding, and π-π interaction. At 303 and 323 K, the highest adsorption capacity was 1017.29 and 1060.45 mg·g^−1^, respectively. Furthermore, when the recycle time was 4, the equilibrium adsorption capacity remained at 665.43 mg·g^−1^, which implied fairly good regeneration performance. The modification provided a simple, environmentally friendly, and economical solution for converting sugarcane bagasse into an efficient adsorbent for MB treatment.

## 1. Introduction

Dyes are utilized in textile, leather, plastics, printing, food, cosmetics, and other industries, while the toxicity and carcinogenicity of dyes in wastewater present a huge challenge to the world [1,2,3,4]. Methylene blue (MB) is one type of phenothiazine cationic dye that is widely utilized in many areas; however, its toxicity, carcinogenicity, and non-biodegradability can harm the human nervous system and eyes and cause gastritis, breathing difficulties, and other illnesses [5,6,7]. Thus, investigating the method of removing MB from wastewater is really crucial.

Physical, chemical, and biological techniques can be employed to remove MB, and the commonly used physical methods are adsorption, nanofiltration, reverse osmosis, ion exchange, and so on [8]. The low cost and ease of application of adsorption make it a promising technology [9]. This process requires an adsorbent material to which contaminants such as MB can be adsorbed. Hydrochar is a common carbon-rich adsorbent prepared by hydrothermal carbonization (HTC) at comparatively moderate temperatures (180–350 °C) and pressure from waste biomass such as sugarcane bagasse [10,11]. Sugarcane bagasse (SCB) is one type of significant residual product in the cane sugar industry, with an estimated 5.26 million tonnes produced worldwide in 2019 [12]. SCB mainly contains cellulose (40–45%), hemicellulose (30–35%), and lignin (20–30%), and polysaccharide constituents are important for framing the carbon skeleton of hydrochar with functional groups [8,13,14,15]. Hydrochar has been reported to have excellent chemical stability, biocompatibility, electrical conductivity, and thermal conductivity, so research about the use of hydrochar as an adsorbent has attracted increasing attention [10,11]. However, hydrochar still requires improvement owing to its low porosity, low specific surface area, and strong binding site deficiency [16,17]. Hydrochar contains abundant hydroxyl groups, which are conducive to offering chemical reactive sites for further covalent functionalization with organic reagents; moreover, adding more oxygen-containing functional groups to hydrochar can increase its adsorption capability [17,18].

Diethylenetriamine contains numerous primary and secondary amino groups, which can react with hydroxyl groups of hydrochar, thereby introducing amino groups. The strong reactivity of amino groups allows them to form reactions with chloroacetic acid to produce carboxylate groups. Introducing amino groups and carboxylate groups is conducive to providing adsorption sites. And the carboxylate groups are negatively charged; hydrochar with carboxylate groups could adsorb MB through electrostatic attraction. Furthermore, introducing polyaminocarboxylate groups through adding these two reagents is simple and economical. Li et al. prepared polyaminocarboxylated modified bamboo hydrochar successfully through adopting these two reagents and confirmed that this method was feasible [17]. This study provides a simple, environmentally friendly, and economic method to transform sugarcane bagasse into polyaminocarboxylated modified hydrochar (ACHC), which has excellent adsorption capacity for MB. The primary goals are (i) to prepare ACHC by introducing polyaminocarboxylate groups through diethylenetriamine and chloroacetic acid; (ii) to characterize ACHC by multiple methods and evaluate its adsorption capacity; and (iii) to explore the adsorption mechanism.

## 2. Results

### 2.1. Characterization

#### 2.1.1. Morphology Analysis

**Figure 1:**
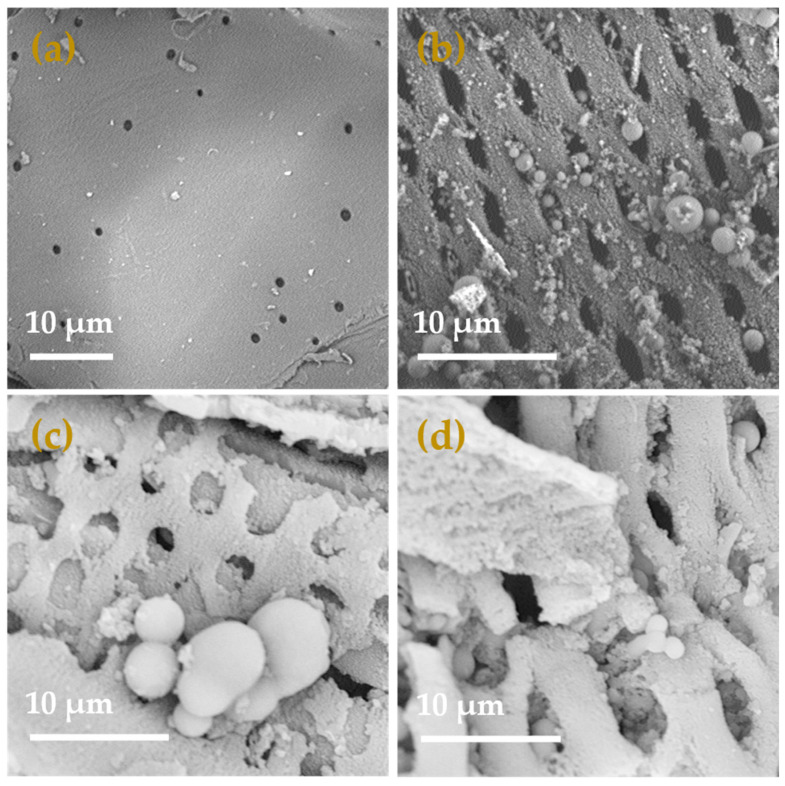
SEM spectra of (**a**) SCB; (**b**) HC; (**c**) ACHC; (**d**) ACHC/MB.

By utilizing scanning electron microscopy (SEM), the morphology of sugarcane bagasse (SCB), sugarcane bagasse hydrochar (HC), polyaminocarboxylated modified hydrochar (ACHC), and polyaminocarboxylated modified hydrochar/methylene blue (ACHC/MB) was examined, and findings are demonstrated in Figure 1. Less porosity and a comparatively smooth and flat surface were seen on SCB (Figure 1a), while HC had a rough surface with open pores evenly distributed (Figure 1b), and there were numerous carbon microspheres of varying sizes randomly distributed on the surface, leading to partial blockage of the pores. The pore blockages were brought on by the hydrocarbon condensation that occurred on the surface during the hydrothermal carbonization (HTC) process [19]. The formation of the spherical structure could be due to the condensation of the solubilized hemicellulose, cellulose, and a tiny amount of lignin during the HTC process with the presence of phosphoric acid [20]. ACHC presented a fluffy and porous surface structure. The pore shape slightly was altered compared with the HC, with round openings rather than elliptical ones observed (Figure 1c), which indicated the modification might further corrode the hydrochar through volatilization and activation [21]. Furthermore, the morphology of the ACHC after the adsorption of MB was visualized (Figure 1d). The external and inner structures of ACHC were occupied by MB to some degree, demonstrating the effective adsorption of MB onto ACHC.

#### 2.1.2. Pore Structure Analysis

**Figure 2:**
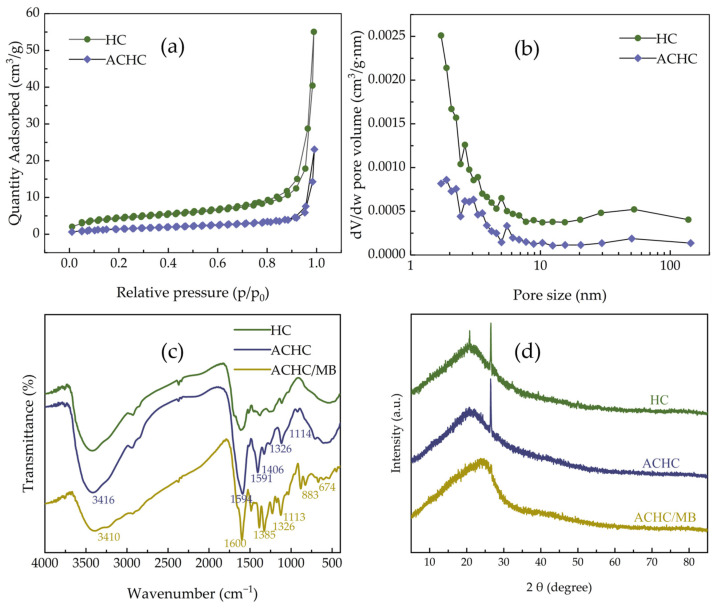
(**a**) N_2_ adsorption-desorption isotherms of HC and ACHC; (**b**) pore size distributions of HC and ACHC; (**c**) FTIR spectrum of HC, ACHC, and ACHC/MB; (**d**) XRD patterns of HC, ACHC, and ACHC/MB.

Porosity of HC and ACHC was analyzed by specific surface area and porosity meter (BET). Figure 2 displays N_2_ adsorption-desorption isotherms together with relevant pore size distribution curves. Type IV isotherms and H_3_ type of hysteresis were displayed (Figure 2a), illuminating the existence of mesopores and irregular slits in HC and ACHC (Figure 2b) [22]. While ACHC showed less N_2_ adsorption capacity, indicating that the modification process impacted surface area and total pore volume, pore size did not significantly change, nevertheless. The results were in agreement with the above-mentioned pore types, and this was further confirmed by calculation in Table 1. Since ACHC had a smaller capacity than HC, its rate of N_2_ adsorption with relative pressure increment increased at a slower pace, which might suggest that the polyaminocarboxylated modification had no improvement in pore structure. The mesoporous structure was not entirely blocked by the grafting of amino and carboxylate groups, which would have been better for the MB adsorption process [16,23].

#### 2.1.3. Functional Group Analysis

HC, ACHC, and ACHC/MB were examined by Fourier transform infrared spectroscopy (FTIR), and the resulting spectra are displayed in Figure 2c. A significant portion of peaks identified in HC was also observed in ACHC, including the wide and strong characteristic band around 3416 cm^−1^ representing the O-H stretching vibrations of hydroxyl or carboxyl groups, which revealed that O-H groups primitively in cellulose, hemicellulose, and lignin were not entirely destroyed in the HTC process [18,24]. Characteristic bands occurring in the region of 1300~1000 cm^−1^ are ascribed to C-O single bonds [25], and distinctive bands at 1594 and 1406 cm^−1^ match with symmetric and asymmetric stretching vibrations in -COOH [26,27]. These characteristic peaks show that both HC and ACHC included a significant number of functional groups that contained oxygen. Because these oxygen-containing functional groups may interact with cationic MB and act as proton donors, ACHC’s adsorption ability may increase with the number increment of oxygen-containing functional groups it contained [18]. It was noteworthy that three unique phenomena occurred following the modification: first, characteristic peak intensity at 3416 cm^−1^ was increased; second, strong peaks emerged at 1594 and 1406 cm^−1^, matching with -COOH groups; third, a new characteristic peak coordinating with C-N groups showed up at 1114 cm^−1^, supposing an N atom had been successfully incorporated on ACHC [28]. The appearance of these different characteristic peaks indicates that the hydroxyl groups of HC had undergone modification with reagents, and the amino groups and carboxylate groups had been successfully introduced. These oxygen-containing groups could provide sufficient adsorption sites for adsorption.

After adsorption of MB by ACHC, the characteristic peaks migrated to 3410, 1591, 1385, 1326, and 883 cm^−1^, indicating that ACHC successfully adsorbed MB [17]. The O-H characteristic peak migrated from 3416 to 3410 cm^−1^, confirming that the hydroxyl groups of ACHC were hydrogen bonded with the N atom in the phenothiazine ring of MB [9,29]. The characteristic peak of C=N migrated from 1589 to 1591 cm^−1^, and C-H stretching vibration shifted from 826 to 883 cm^−1^, suggesting π-π interaction between adsorbent and adsorbate [17]. C-N and symmetric -CH_3_ stretching vibrations of -N(CH_3_)^2+^ migrated from 1389 and 1319 to 1385 and 1326 cm^−1^ respectively, confirming electrostatic interaction between ACHC and MB [9].

#### 2.1.4. Structure Property Analysis

Figure 2d displays X-ray diffractometer (XRD) patterns of HC, ACHC, and ACHC/MB. In HC and ACHC, an obvious diffraction peak around 2θ of 23.8° corresponding to the (002) crystal plane was observed, supposing the production of graphitic carbon structures [30,31]. Modification merely modulated the structural properties and did not affect the crystallinity, as evidenced by the fact that the position and intensity of the diffraction peaks did not vary appreciably [32]. After adsorption of MB onto ACHC, the diffraction peak at 2θ = 23.8° shifted to the right, and the peak width increased, supposing the crystallinity was changed after adsorption of MB by ACHC, which meant that MB was successfully adsorbed by ACHC.

#### 2.1.5. Chemical Composition Analysis

**Figure 3:**
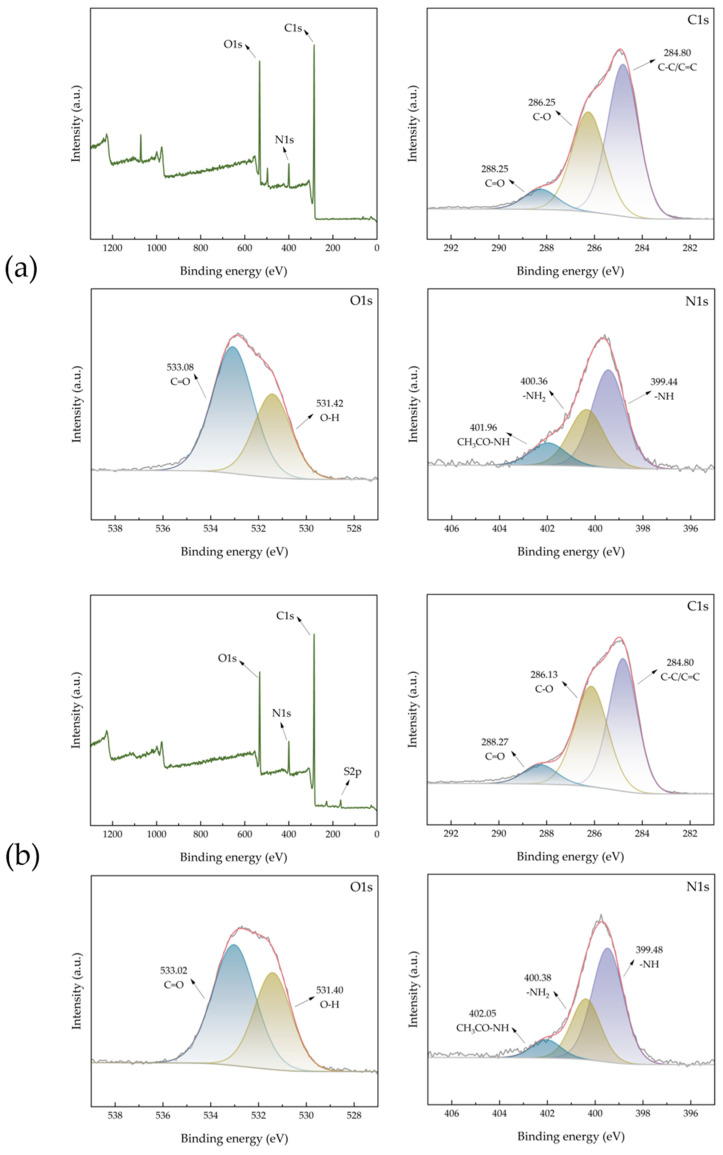
The XPS spectra of (**a**) ACHC and (**b**) ACHC/MB.

Chemical compositions of ACHC were analyzed by X-ray photoelectron spectroscopy (XPS) (Figure 3a). Three peaks at 284.80, 286.25, and 288.25 eV were identified in the C1s spectrum of ACHC, coordinating with C-C/C=C, C-O, and C=O [33]. ACHC was rich in oxygen-containing functional groups, as evidenced by the O1s spectrum, which could be deconvoluted into two peaks at 531.42 and 533.08 eV, reflecting O-H and C=O [34]. This observation aligned with the FTIR analysis conclusion. Groups that concluded oxygen were able to serve as negatively charged active adsorption sites, and higher adsorption was produced by electrostatic interaction between oxygen-containing groups and MB [35]. The spectrum of N1s can be fitted to three peaks, namely -NH (399.44 eV), -NH_2_ (400.36 eV), and CH_3_CO-NH (401.96 eV), demonstrating that the N atom had been successfully incorporated on ACHC [36], which conformed well with the results shown in FTIR analysis. The addition of groups containing nitrogen may be responsible for the increase in adsorption capability. Furthermore, due to the high content of C=O in O1s and the high content of -NH in N1s (Figure 3), these two functional groups were chosen as the representative functional groups in the density functional theory (DFT) analysis later.

As shown in Figure 3b, after the adsorption of MB, a new elemental peak emerged at 164.94 eV (S2p), which demonstrated that ACHC had successfully adsorbed MB [37]. And after the adsorption of MB, there were changes in the binding energies of C1s and O1s, suggesting that the oxygen-containing functional groups had participated in the adsorption, which was consistent with the results of FTIR [37].

### 2.2. Effects of pH

**Figure 4:**
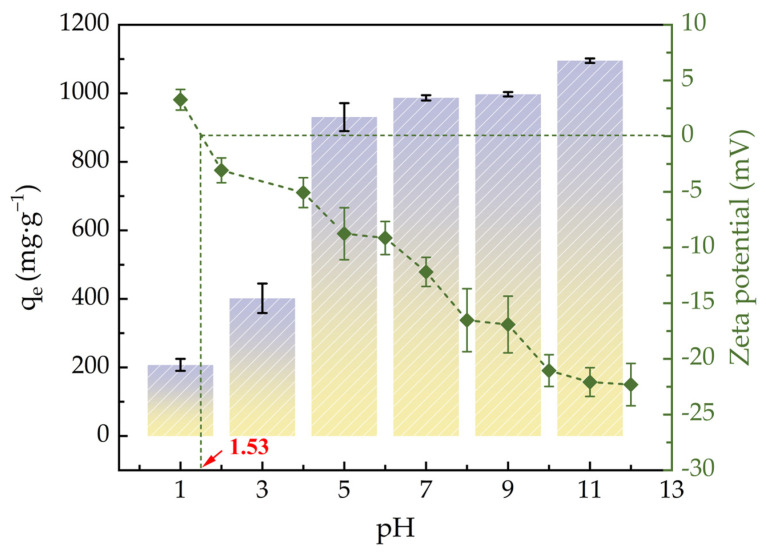
The pH’s effects on zeta potential and adsorption capacity (*q_e_* means adsorption amount at adsorption equilibrium) of ACHC (bar graph refers to left axis, line graph refers to right axis).

Figure 4 illustrates how the initial pH value affects the zeta potential and ACHC’s adsorption capacity for MB. ACHC’s adsorption capacity was relatively low between pH of 1.0–3.0; it increased sharply when pH increased to 5.0; it increased slowly between pH of 7.0–11.0; and it reached its maximum when pH was 11.0. With the increment of pH value, the zeta potential of ACHC decreased continuously, and the electronegativity of ACHC increased constantly. In aqueous solution, MB may exist in the cationic form (MB^+^) or exist as undissociated molecules (MB^0^). According to experimental results, the pH_pzc_ of ACHC was about 1.5. When pH < pH_pzc_, MB^0^ was the main part of MB and the ACHC surface was positively charged, which would generate electrostatic repulsion with tiny dissociated MB^+^, hindering the adsorption of MB by ACHC, and excess H^+^ would compete with MB^+^ for adsorption sites, which was unfavorable to the adsorption of MB by ACHC [38]. Meanwhile, phenothiazine’s N atoms in MB were easily protonated at low pH levels, which hindered adsorption by hydrogen bonding [29]. When pH > pH_pzc_, functional groups on the ACHC surface were prone to deprotonation so that ACHC was negatively charged and MB almost existed as MB^+^, which promoted the electrostatic attraction between ACHC and MB, promoting ACHC to adsorb MB [38]. Simultaneously, there was a higher probability of hydrogen bonding between phenothiazine’s N atoms in MB and the C-OH in ACHC [5].

### 2.3. Adsorption Capacity Comparison

**Figure 5:**
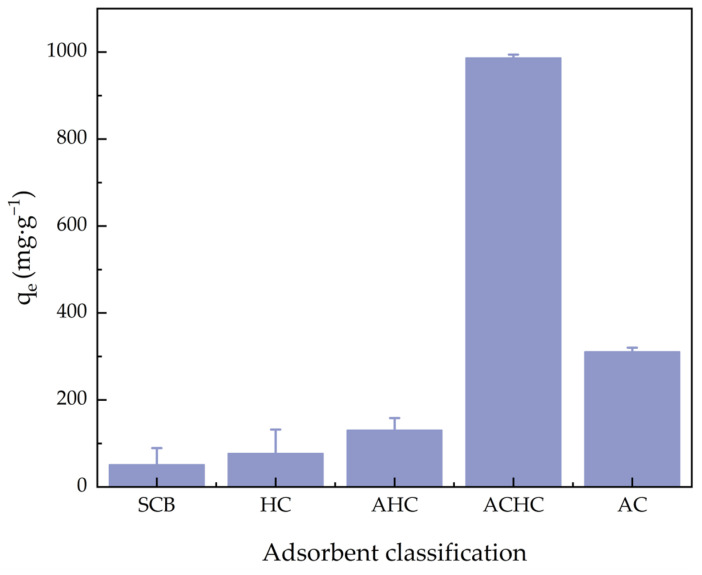
Comparison of adsorption capacity for MB of SCB, HC, AHC, ACHC, and AC.

Figure 5 shows the MB adsorption capacity comparison of SCB, HC, activated hydrochar (AHC), ACHC, and activated carbon (AC). AC is a kind of commercial adsorbent, and we used AC to compare adsorption capacity with our materials. The adsorption capacities for MB were 51.36, 77.23, 130.68, 986.73, and 310.87 mg·g^−1^, respectively. The MB adsorption ability of HC was slightly higher than SCB because the aromaticity and porosity of HC were improved during the HTC process in a phosphoric acid medium. The MB adsorption ability of AHC was slightly higher than HC due to the NaOH activation. And the adsorption capacity of ACHC was much higher than HC, mainly because of the introduction of functional groups, demonstrating that modification was greatly necessary. Furthermore, the adsorption capacity of ACHC was also much higher than AC, confirming that adsorbent ACHC can outperform commercial activated carbon. Table 2 summarizes MB’s *q_m_* (the maximum equilibrium adsorption capacity) on different adsorbents, and ACHC’s maximum adsorption capability was top-ranked.

**Figure 6:**
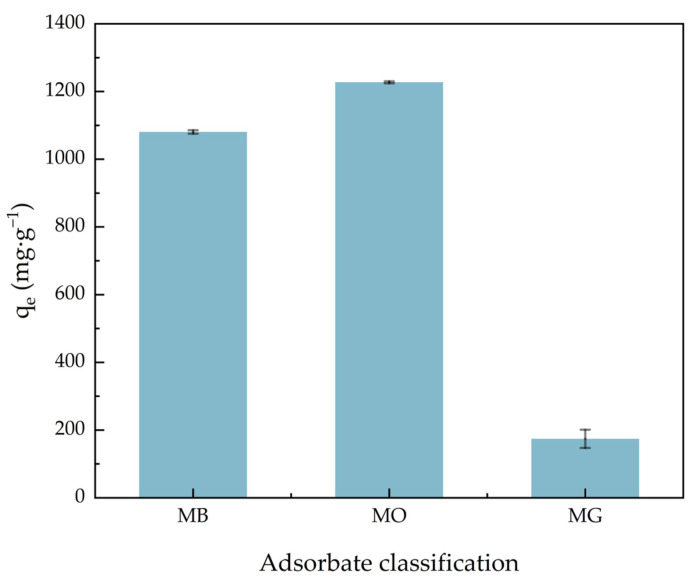
Comparison of ACHC adsorption ability for MB, MO, and MG.

Figure 6 shows the comparison of ACHC adsorption capacity on different dyes. In the multiple dye system, which contained three different dyes at the same time, the adsorption capacity of methylene blue (MB), methyl orange (MO), and malachite green (MG) was 1080.46, 1227.29, and 174.20 mg·g^−1^, respectively. The π-π stacking between MB and MO may participate in the adsorption of MB, and the MB molecules adsorbed by the adsorbent may become new adsorption sites for MO adsorption due to the synergistic effect [43,44]. Furthermore, our modification introduced many carboxylate groups and amino groups, and a predecessor’s study showed that carboxylate groups were beneficial for the adsorption of MB and the amino groups were beneficial for the adsorption of MO [45,46]. And the poor adsorption capacity of MG may be relevant to its big molecular weight, because small dye molecules were more easily able to enter the pores of the adsorbent [47].

### 2.4. Adsorption Isotherm Models

**Figure 7:**
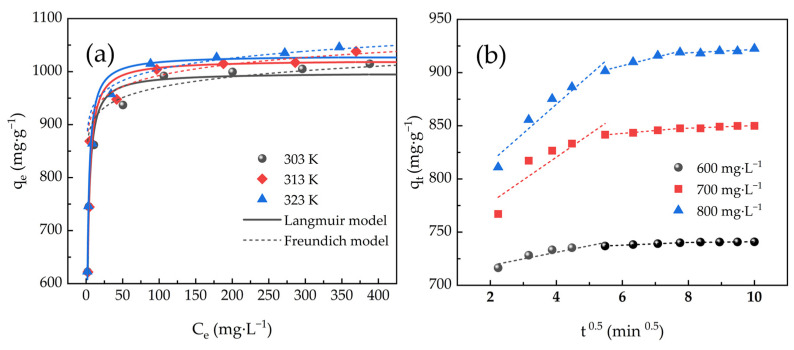
(**a**) Langmuir and Freundlich fitting curves of ACHC/MB and (**b**) linearity of IPD model for adsorption.

Fitting curves of adsorption at three distinct temperatures are depicted in Figure 7a. As the value of *C_e_* increased, *q_e_* increased rapidly, then slowly, and eventually reached a plateau. As temperature rose, *q_m_* rose as well, indicating that adsorption was essentially endothermic. Whereas the Freundlich model simulated heterogeneous multilayer adsorption behavior, the Langmuir model imitated homogeneous monolayer adsorption behavior [48].

The equation of the Langmuir isotherm model is [49]:

(1) $$q_{e}=\frac{q_{m}\cdot K_{L}\cdot C_{e}}{1+K_{L}\cdot C_{e}}$$

where *q_e_* (mg·g^−1^) means adsorption amount at adsorption equilibrium; *q_m_* (mg·g^−1^) means the maximum equilibrium adsorption capacity; *K_L_* (L·mg^−1^) stands for the adsorption constant of the Langmuir isotherm model; *C_e_* (mg·g^−1^) represents residual dye concentration at equilibrium adsorption.

The equation of the Freundlich isotherm model is [49]:

(2) $$q_{e}=K_{F}\cdot C_{e}^{\frac{1}{n}}$$

where *K_F_* (mg·g^−1^) means constant of Freundlich isotherm model; *n* stands for constant that is used to assess how adsorbent and adsorbate interact.

Table 3 displays parameters of the adsorption isotherm model, and parameters were calculated from fitting curves. The Langmuir model’s correlation coefficients (R^2^) were greater than the Freundlich model’s R^2^, and *q_m_* of MB adsorbed by ACHC corresponding to the Langmuir model were 1017.29, 1048.22, and 1060.45 mg·g^−1^ respectively when at 303, 313, and 323 K, which were consistent with actual experimental data. Therefore, the Langmuir model characterized adsorption more accurately than Freundlich did, supposing it was homogeneous monolayer adsorption [29].

### 2.5. Adsorption Mass Transfer Model

Intra-particle diffusion (IPD) model of MB adsorbed by ACHC was presented in Figure 7b, which describes a multi-level linear relationship deviating from the origin, supposing adsorption was involved in multiple mechanisms.

The IPD model derived by Weber and Morris is [50]:

(3) $$q_{t}=K_{IPD}t^{0.5}+c$$

where *q_t_* (mg·g^−1^) represents adsorption capacity at time t; *K_IPD_* (mg·g^−1^·min^−0.5^) means IPD rate constant; *c* (mg·g^−1^) stands for linear graph intercept of *q_t_* versus *t*^0.5^.

Table 4 displays parameters of the IPD model used for adsorption. At the three distinct MB initial concentrations, the IPD model’s R^2^ were all high (R^2^ > 0.644), indicating that IPD would affect adsorption rate. Multiple stages governed the mass transfer process, as evidenced by the multilinear relationship deviating from the origin that the IPD model exhibited. The concentration difference between the substrate solution and the adsorbent surface drove the first step of external diffusion, where the adsorbate penetrated the liquid layer around the adsorbent. Adsorbent dispersed inside its pores during the second step, known as internal diffusion. Adsorbate bonded to its active site in the third step, which was the last equilibrium stage. The rate-limiting process was not limited to internal diffusion alone [38].

### 2.6. Adsorption Thermodynamics Model

Thermodynamic parameters include Gibbs free energy (Δ*G*), enthalpy (Δ*H*), entropy (Δ*S*), and so on. Van’t Hoff’s equation is used to calculate thermodynamic parameters.

Van’t Hoff’s equation is [51]:

(4) $$K=\frac{q_{e}}{C_{e}}$$

(5) ΔG=−RTlnK

(6) $$\ln K=\frac{-\Delta H}{RT}+\frac{\Delta S}{R}$$

where *K* means adsorption distribution coefficient; ∆*G* (kJ·mol^−1^) stands for Gibbs free energy; *R* (8.314 J·mol^−1^·K^−1^) represents universal gas constant; *T* (K) denotes absolute temperature; ∆*H* (kJ·mol^−1^) is enthalpy; and ∆*S* (kJ·mol^−1^·K^−1^) is entropy.

Thermodynamic parameters are summarized in Table 5. A positive ∆*H* value (5.81 kJ·mol^−1^) suggested adsorption was facilitated by rising temperature; that is, adsorption of MB by ACHC was endothermic, which was consistent with the isotherm model conclusion; a positive ∆*S* value (13.38 J·mol^−1^·K^−1^) suggested strong randomness of the solid-liquid interface in adsorption; and a negative ∆*G* value (−4.05~4.70 kJ·mol^−1^) suggested adsorption was spontaneous [16]. Physical adsorption occurs when Δ*G* is between −20 and 0 kJ·mol^−1^, and chemical adsorption occurs between −400 and −80 kJ·mol^−1^ [52]. The ∆*G* value obtained from the adsorption thermodynamic analysis was between −4.70 and −4.05 kJ·mol^−1^, indicating that adsorption was physical. We noticed that the nonlinear form of the thermodynamic model was better than its linear form when it was used for adsorption analysis; however, the difference in the magnitude of the thermodynamic parameters (∆*H* and ∆*S*) calculated between these two forms was not remarkable [53].

### 2.7. Density Functional Theory (DFT) Calculations

**Figure 8:**
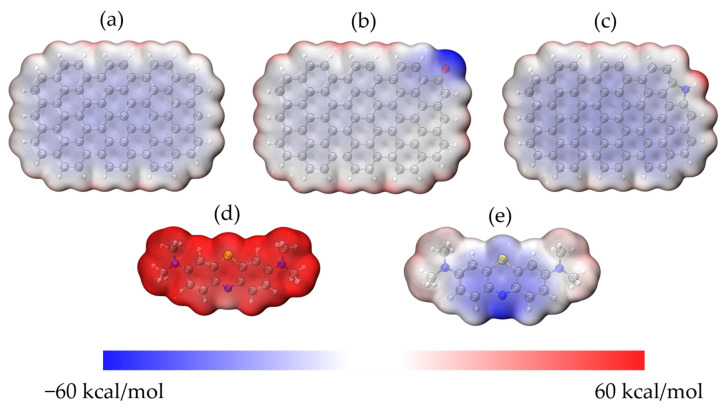
ESP surfaces of (**a**) graphene; (**b**) carbonyl-graphene; (**c**) pyrrolic nitrogen-graphene; (**d**) MB^+^ and (**e**) MB^0^. Grey: carbon, white: hydrogen, red: oxygen, blue: nitrogen, and yellow: sulfur.

To see how the molecules of MB and ACHC interacted, DFT calculations were performed. The 24-ring graphene structure was employed to represent HC [54], and functional groups on the graphene structure were proposed based on FTIR and XPS study results as discussed earlier. Oxygen-containing functional group C=O and nitrogen-containing group pyrrolic N were the two main functional groups identified; therefore, the pristine 24-ring graphene and that with C=O and pyrrolic N groups were used in this study. A 24-ring graphene represented HC, while both 24-ring graphene with C=O and 24-ring graphene with pyrrolic N represented ACHC. And the surface electrostatic potential (ESP) of the models was provided based on the geometry-optimized structures (Figure 8). As shown in Figure 8, the electrical distribution of molecules was represented and reaction sites were predicted, reflecting the electrostatic interactions between molecules [42,55]. Electrostatic potential (ESP) values increased from −60 to 60 kcal/mol while the color changed from blue to red, signifying positively and negatively charged areas of the molecule [42,56]. The negatively charged C=O site and the slightly positively charged pyrrolic N site on the surface of the graphene ring could be the active sites for adsorption of the ionized MB molecules (MB^+^) and the unionized MB molecules (MB^0^), respectively. MB ionization in aqueous solution was a spontaneous process that arose from the electrophilic -N(CH_3_)_2_ groups that existed in the MB^0^ and MB^+^, was the dominant form in our batch adsorption experiments; hence, the positively charged MB^+^ was used in the following calculations [42,55,57]. There was an electrostatic attraction between MB and pristine graphene/modified graphene, because the electron distribution in MB molecule created a positively charged molecular surface and pristine graphene/modified graphene formed a negatively charged molecular surface [54,58]. Notably, the oxygenated functional group (C=O) formed a preferentially negative surface, which enhanced the electrostatic attraction. FTIR analysis mentioned above also demonstrated electrostatic attraction.

**Figure 9:**
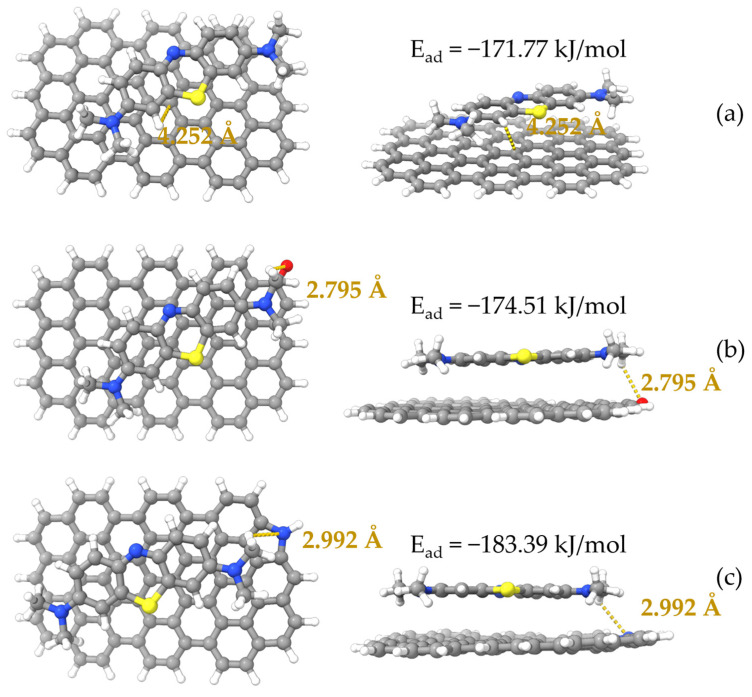
Optimized geometries of MB adsorbed on different graphene surfaces. (**a**) MB/graphene; (**b**) MB/carbonyl-graphene; and (**c**) MB/pyrrolic nitrogen-graphene. Grey: carbon, white: hydrogen, red: oxygen, blue: nitrogen and yellow: sulfur.

Optimized configurations are listed in Figure 9, with binding energy or adsorption energy calculated. With an affinity of only −171.77 kJ/mol, MB had the lowest affinity to pristine graphene. Graphene grafted with the two functional groups showed improved interactions with MB in different degrees [54], with the pyrrolic N functionalized graphene providing the best adsorption of MB, of which the adsorption energy was −183.39 kJ/mol. The improved adsorption performance of oxygen-containing and nitrogen-containing functional groups modified graphene explained that the polyaminocarboxylated modification had improved adsorption capacity [49]. And even the pristine graphene structure also showed satisfactory adsorption energy. The strong polarization effect of π-electron density may be the cause of the strong interaction between pristine graphene/modified graphene [59,60]. If adsorption energy was lower than −30 kJ/mol, the interactions between the pristine graphene/modified graphene and MB can be classified as physical adsorption, and the interactions can be regarded as chemisorption behavior if adsorption energy was higher than −50 kJ/mol [49,61]. Adsorption energy was completely negative and lower than −50 kJ/mol for pristine and modified graphene, indicating that the interactions were physical adsorption, which was consistent with adsorption thermodynamics analysis.

Figure 9 also shows the results of hydrogen bonding calculation for the model after MB adsorption. Functional groups (C=O, pyrrolic N) of graphene had hydrogen bond distances of 2.795 Å and 2.992 Å, supposing adsorbate could be adsorbed onto modified graphene because of hydrogen bonding [42]. The FTIR analysis showed that there was hydrogen bonding between ACHC’s hydroxyl groups and the N atom in the phenothiazine ring of MB [9,29]. It is worthwhile to mention that graphite phases may be generated by HTC of glucose, even if π-π stacking interactions between MB and graphene were not calculated [58,59,60,61,62]. It is possible to anticipate the occurrence of π-π stacking between aromatic phases in ACHC and MB based on characteristics of ACHC precursor material [62], agreeing with the FTIR analysis above.

### 2.8. Adsorption Mechanisms

SEM analysis displayed that MB could be successfully adsorbed by ACHC. Pollutant removal performance may be improved by the rough surface, adequate cracks, channels, and pores, as well as the loaded functional groups [63]. The BET study showed that polyaminocarboxylated modification had no improvement on the pore structure. But the mesoporous structure was not completely clogged by grafting functional groups, which implied that modification might lead to higher adsorption capacity. FTIR analysis showed that adsorption of MB by ACHC involved hydrogen bonding and π-π interaction together with electrostatic attraction. After adsorption, migration of the C-H characteristic peak indicated the existence of hydrogen bond association. The migration of the C=N characteristic peak and the migration of the C-H stretching vibration indicated the existence of π-π interaction, and the stretching vibrations of C-N and symmetric CH_3_ indicated the presence of electrostatic attraction. XRD analysis showed that crystallinity changed after adsorption of MB onto ACHC, illustrating that ACHC had successfully adsorbed MB. XPS analysis demonstrated that ACHC revealed a high concentration of oxygen-containing groups and nitrogen-containing groups; it also revealed the successful adsorption for MB.

Adsorption isotherm model analysis revealed that adsorption followed the Langmuir model, which supposed adsorption was a homogeneous monolayer. And the adsorption was affected by the rate of IPD. Thermodynamic analysis suggested adsorption was physical, endothermic, and spontaneous, and randomness of the solid-liquid interface was strong. The pH analysis showed that electrostatic repulsion between tiny dissociated MB^+^ and positively charged ACHC and competition between tiny dissociated MB^+^ and excessive H^+^ at adsorption sites would hinder the adsorption of MB by ACHC, while electrostatic attraction between MB^+^ and negatively charged ACHC would facilitate adsorption. And DFT analysis showed that there was both physical and chemical adsorption between ACHC and MB.

### 2.9. Adsorbent Regeneration

**Figure 10:**
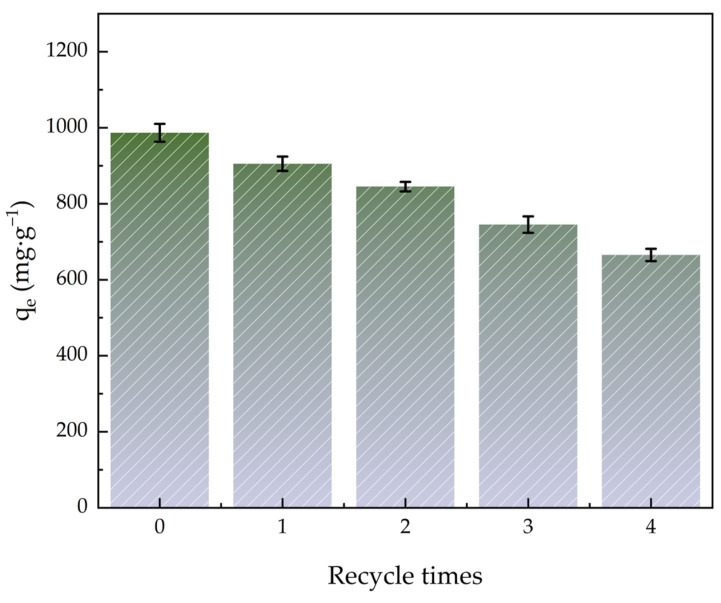
Effect of quantity of regeneration cycles on adsorption efficiency.

Figure 10 shows how the quantity of ACHC regeneration cycles affected MBs adsorption efficiency. Adsorption capacity was still high after the adsorption-desorption cycle, and it remained high at 665.43 mg·g^−1^ after five adsorption-desorption cycles, indicating that ACHC could be used to remove MB repeatedly and it had a great recovery capacity. Perhaps it was because MB did not bind to the strongest binding site, and the porous structure of ACHC provided space for MB desorption diffusion [64].

## 3. Materials and Methods

### 3.1. Materials

Sugarcane bagasse (SCB) was sourced from Guangxi Fengtang Biochemical Co., Ltd. (Liuzhou, China). Phosphoric acid, sodium hydroxide, hydrochloric acid, methylene blue (MB), and malachite green (MG) were obtained from Sinopharm Chemical Reagent Co., Ltd. (Shanghai, China). Anhydrous ethanol was procured from Tianjin Kemiou Chemical Reagent Co., Ltd. (Tianjin, China). Epichlorohydrin, diethylenetriamine, and chloroacetic acid were supplied by Shanghai Macklin Biochemical Technology Co., Ltd. (Shanghai, China). Activated carbon (AC) was sourced from Dongguan Hongsheng Activated Carbon Co., Ltd. (Dongguan, China). Methyl orange (MO) was obtained from Tianjin Damao Chemical Reagent Factory (Tianjin, China).

### 3.2. Preparation of Sugarcane Bagasse Hydrochar (HC)

SCB powder and a 1.5 mol·L^−1^ phosphoric acid solution were mixed in a Teflon-lined autoclave with a mass ratio of 1:19. The reactor was placed in a muffle furnace and reacted at 513 K for 10 h. After reaction, the reactor was taken out and cooled to room temperature, then the mixture was filtered and washed with deionized water and anhydrous ethanol until the extracted filtrate was nearly colorless; finally, the solid was dried to constant weight at 378 K and was denoted as HC. The HC was further ground and screened by a 100-mesh sieve (diameter = 0.15 mm) and was stored in a brown glass bottle for further use.

### 3.3. Preparation of Polyaminocarboxylated Modified Hydrochar (ACHC)

**Figure 11:**
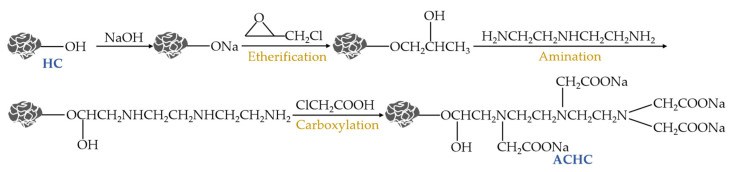
Synthetic route of ACHC.

ACHC was synthesized referring to the method described in previous reports from predecessors’ literature, as Figure 11 [17]. HC (2.5 g) was put in 50 mL of 1.5 M NaOH solution and then agitated at 150 rpm for 2 h at 298 K, followed by vacuum filtration, repeated washing, and drying at 353 K overnight to constant weight. The obtained product was ground using an agate mortar, passed through a 100-mesh sieve, sealed, and stored in a brown bottle, and was denoted as Activated hydrochar (AHC). Three steps, including etherification, amination, and carboxylation, were then performed to obtain ACHC. Specifically, 20 g AHC was mixed with 100 mL epichlorohydrin and then reacted under vigorous stirring at 353 K for 4 h, filtered, washed, and dried at 333 K to steady mass. The obtained product was denoted as etherified hydrochar. This was further subjected to the amination treatment, where etherified hydrochar was added to the mixture of water/diethylenetriamine (*v*/*v* of 1:1) and agitated at 333 K for 4 h, filtered, washed, and dried at 333 K to stable mass and designated as aminated hydrochar. The aminated hydrochar was then further subjected to the carboxylation procedure, where 15 g of aminated hydrochar and 25 g of sodium hydroxide were mixed with 50 mL of deionized water and 200 mL of anhydrous ethanol, stirred for 10 min at ambient temperature. This was followed by adding a solution containing 60 g chloroacetic acid and 100 mL anhydrous ethanol dropwise within 60 min and reacting under stirring at 333 K for 4 h. The reaction system’s pH was kept above 9.0 by adding 0.1 M sodium hydroxide. After reaction, the product was filtered, washed, and dried at 333 K to a consistent mass, thereby obtaining polyaminocarboxylated hydrochar (ACHC).

### 3.4. Characterization of Carbon Materials

Structural properties were measured by scanning electron microscopy (SEM; Tescan Mira Lms, Tescan, China) at an acceleration voltage of 5.00 kV. A specific surface area and porosity meter (BET; ASAP 2460, Micromeritics, USA) was employed to generate N_2_ adsorption-desorption isotherms to study specific surface area, pore volume, as well as pore size. Surface functional groups were characterized by using Fourier transform infrared spectroscopy (FTIR; TENSOR II, Bruker, Germany) at resolution of 6 cm^−1^. Crystallinity was analyzed by X-ray diffractometer (XRD) with Cu-Kα as the radiation source in a scanning range of 2θ = 5–85°. Composition and chemical state were studied by X-ray photoelectron spectroscopy (XPS; ESCALAB 250Xi, ThermoFisher, USA). The data obtained was calibrated against the standard C1s binding energy set at 284.80 eV. Zeta potential at different pH was determined by using a zeta potential analyzer (zeta potential; NanoBrook Omni, Brookhaven, USA).

### 3.5. Batch Adsorption Experiments

To a 100 mL conical flask, 50 mL of MB of specified concentration was added. The dosage of ACHC in each bottle was 40 mg. An orbital shaker was used to shake the sealed conical flasks at a fixed temperature for a set amount of time while maintaining an invariable speed of 150 rpm in an incubator. After adsorption, ACHC was filtered with a 0.45 μm microporous filter membrane. Adsorption efficiency was determined by recording absorbance at 665 nm of filtrate, and MB concentration in filtrate was calculated based on the MB standard curve.

Effects of pH: Ten portions of 50 mL of 1000 mg·L^−1^ MB were fabricated, and the pH was reset to 2.0, 3.0, 4.0, 5.0, 6.0, 7.0, 8.0, 9.0, 10.0, and 11.0 using 0.1 M sodium hydroxide or hydrochloric acid. To the flasks, 40 mg ACHC was added, and the flask was oscillated at 303 K for 24 h, followed by determining MB concentration in the filtrate. A zeta potential analyzer was used to determine the zeta potential of the adsorbent. Three parallel experiments were conducted.

Adsorption comparison among adsorbents of SCB, HC, AHC, ACHC, and AC: To 100 mL conical flasks, 50 mL MB solution of 1000 mg·L^−1^ was added, respectively. The pH was reset to 7.0 using 0.1 M sodium hydroxide or hydrochloric acid. The dosage of adsorbent in each bottle was 40 mg. An orbital shaker was used to shake the sealed conical flasks at 303 K for 24 h at 150 rpm, followed by filtering the solution with a 0.45 μm filter membrane. Adsorption efficiency was determined by recording absorbance at 665 nm of filtrate, and concentration was calculated based on the standard curve. Three parallel experiments were conducted.

Adsorption comparison among adsorbates of MB, MO, and MG: To a 100 mL conical flask, 50 mL solution was added. The MB, MO, and MG concentrations in the solution were 1000 mg·L^−1^, respectively. The pH was reset to 7.0 using 0.1 M sodium hydroxide or hydrochloric acid. The dosage of adsorbent ACHC in each bottle was 40 mg. An orbital shaker was used to shake the sealed conical flasks at 303 K for 24 h at 150 rpm, followed by filtering the solution with a 0.45 μm filter membrane. Adsorption efficiency was determined by recording absorbance at 665 nm (MB), 464 nm (MO), and 620 nm (MG) of filtrate, and concentration was calculated based on standard curves. Three parallel experiments were conducted.

Adsorption performance: The two following formulas were used to calculate adsorption capacity at adsorption equilibrium and time t [49].

(7) $$q_{e}=\frac{(C_{0}-C_{e})V}{m}$$

(8) $$q_{t}=\frac{(C_{0}-C_{t})V}{m}$$

where *q_e_* (mg·g^−1^) means adsorption amount at adsorption equilibrium; *C*_0_ (mg·g^−1^) stands for dye starting concentration; *C_e_* (mg·g^−1^) means residual dye concentration at equilibrium adsorption; *V* (L) denotes dye volume; *m* (g) is adsorbent mass; *q_t_* (mg·g^−1^) means adsorption capacity at time t; and *C_t_* (mg·g^−1^) is residual dye concentration at time t (min).

Adsorption isotherm study: 50 mL MB of 500, 600, 700, 800, 900, 1000, 1100, and 1200 mg·L^−1^ was placed in a flask. The pH was reset to 7.0, and 40 mg ACHC was added. The flask was oscillated at 303, 313, and 323 K for 24 h and filtered. MB concentration in filtrate was measured. Adsorption capacity was calculated when the adsorption reached equilibrium.

The adsorbent regeneration behavior was also observed. For 24 h, at 303 K, ACHC (40 mg) was added to MB (50 mL), which had an initial pH of 7.0 and a starting concentration of 1 mg·mL^−1^. Adsorbent was repeatedly eluted with a 0.1 M hydrochloric acid solution and then dried after being repeatedly washed and filtered with 0.1 M sodium hydroxide and deionized water. Three parallel experiments were conducted.

### 3.6. Density Functional Theory (DFT) Calculations

Gaussian 16, A03, was the software program used for all DFT computations [65]. With the PCM solvation model of water and the B3LYP functional and 6–31 G* base, geometry optimization calculations were carried out with Grimme dispersion corrections (GD3BJ). The B3LYP functional and a bigger 6–311 G* base with the SMD solvation model of water, containing Grimme dispersion corrections (GD3BJ), were then used as the foundation for singlet point energy calculations. The Multiwfn 3.8 (dev) program assisted in achieving the ESP [66].

## 4. Conclusions

The ACHC prepared by activation, etherification, amination, and carboxylation of SCB after HTC treatment had extraordinary ability to adsorb MB. SEM analysis demonstrated that ACHC provided more adsorption sites for MB adsorption when compared with HC. Although BET analysis demonstrated that modification did not improve the pore structure, FTIR and XPS analysis showed that modification introduced plenty of oxygen- and nitrogen-containing groups, which were very beneficial for improving adsorption capacity. XRD analysis confirmed that MB was successfully adsorbed by ACHC. According to adsorption models, the adsorption was endothermic spontaneous homogeneous monolayer adsorption dominated by both chemical adsorption and physical adsorption with intra-particle diffusion. DFT calculations revealed that C=O and pyrrolic N modification improved adsorption energy and the adsorption involved electrostatic attraction, hydrogen bonding, and π-π interaction, conforming with characterization results. ACHC not only had high adsorption capacity but also displayed good regeneration performance, indicating that ACHC might be utilized as an excellent adsorbent for adsorbing MB in wastewater.

## Figures and Tables

**Table 1:** BET surface areas, total pore volumes, and average pore sizes of HC and ACHC.

Samples	Surface Area (m^2^·g^−1^)	Total Pore Volume (cm^3^·g^−1^)	Average Pore Size (nm)
HC	7.845	0.060	6.869
ACHC	5.394	0.035	6.592

**Table 2:** Comparison of adsorption capacity for MB with reported results in literature.

Adsorbents	Feedstock	Adsorption Parameters	Adsorption Capacity *q_m_* (mg·g^−1^)	References
MAHC	Bamboo	C_0_ = 100–900 mg·L^−1^, m = 40 mg, T = 303–323 K	657.89 (303 K)	[16]
ACHC	Bamboo	C_0_ = 400–1200 mg·L^−1^, m = 40 mg, T = 303–323 K	1238.66 (303 K)	[17]
MHC	Olive wood	C_0_ = 2–500 mg·L^−1^, m = 20 mg, T = 298 K	257.143 (298 K)	[39]
H-G9	Glucose	C_0_ = 10–45 mg·L^−1^, m = 15 mg, T = 298–328 K	332.46 (298 K)	[40]
FHC	Pomegranate peel	C_0_ = 5–100 mg·L^−1^, m = 100 mg, T = 298 K	556.33 (298 K)	[41]
PMHC-KOH	Persimmon peel, montmorillonite	C_0_ = 50–500 mg·L^−1^, m = 30 mg, room temperature	278.41 (room temperature)	[42]
ACHC	Sugarcane bagasse	C_0_ = 500–1200 mg·L^−1^, m = 40 mg, T = 303–323 K	1017.29 (303 K)	this study

**Table 3:** Isotherm model parameters.

Temperature (K)	*q_e,exp_* (mg·g^−1^)	Langmuir Model			Freundlich Model		
		*q_m_* (mg·g^−1^)	*b*	R^2^	*K_L_* (L·mg^−1^)	1/*n*	R^2^
303	1014.68	1017.29	0.4115	0.9999	649.76	0.0825	0.8803
313	1038.12	1048.22	0.3705	0.9997	653.01	0.0854	0.8258
323	1056.93	1060.45	0.3534	0.9995	659.58	0.0881	0.8929

**Table 4:** IPD model parameters.

Concentration (mg·L^−1^)	*K_IPD_* (mg·g^−1^·min^1/2^)	*c*	R^2^
600	2.3614	720.69	0.7193
700	7.3537	787.64	0.6446
800	11.7640	820.65	0.7972

**Table 5:** Thermodynamic model parameters.

Temperature (K)	Δ*G* (kJ·mol^−1^)	Δ*H* (kJ·mol^−1^)	Δ*S* (J·mol^−1^·K^−1^)	R^2^
303	−4.05	5.81	32.53	0.9974
313	−4.39			
323	−4.70			

## Data Availability

The original contributions presented in this study are included in the article. Further inquiries can be directed to the corresponding author.
